# Meta-analysis and trial sequential analysis of acupoint catgut embedding in the treatment of ulcerative colitis: Acupoint catgut embedding treating ulcerative colitis meta-analysis

**DOI:** 10.1097/MD.0000000000030945

**Published:** 2022-11-25

**Authors:** Yunfeng Yu, Manli Zhou, Yaling Tong, Shuang Yin, Gang Hu, Weixiong Jian, Ying Zhu

**Affiliations:** a The First Affiliated Hospital of Hunan University of Chinese Medicine, Changsha, China; b College of Chinese Medicine, Hunan University of Chinese Medicine, Changsha, China; c Acupuncture and Rehabilitation Clinical School of Medicine, Guangzhou University of Chinese Medicine, Guangzhou, China.

**Keywords:** acupoint catgut embedding, meta-analysis, trial sequential analysis, ulcerative colitis

## Abstract

Although anti-inflammatory and immunomodulatory measures have delayed the progression of ulcerative colitis (UC) to a certain extent, the adverse drug reactions and recurrence after recovery still trouble clinicians. Acupoint catgut embedding is a possible alternative strategy for the treatment of UC, but its clinical efficacy remains controversial. Therefore, this study systematically evaluated the clinical efficacy and safety of acupoint catgut embedding compared with conventional western medicine in the treatment of UC.

**Methods:**

VIP, Wanfang, China National Knowledge Infrastructure, China Biology Medicine, PubMed, Embase, Web of Science, the Cochrane Library databases were searched. And the publication time of the literature was limited from the time that the database was established to February 2022. Two researchers independently screened the literature, extracted data, and assessed risk of bias as required. Meta-analysis was performed with Revman 5.3. Trial sequential analysis (TSA) was performed with TSA 0.9.5.10 Beta. Publication bias was assessed by Stata 15.0. And evidence quality was appraised with GRADEpro3.6.

**Results:**

A total of 10 studies were listed, with a total sample size of 782 cases. Meta-analysis showed that compared with conventional western medicine, acupoint catgut embedding can effectively improve the total effective rate of clinical symptoms (relative risk [RR] = 1.16, 95% confidence interval [CI] = [1.09,1.24], *P *< .00001), endoscopic total effective rate (*RR* = 1.16, 95%*CI* = [1.08,1.25], *P* < .0001), clinical symptom cure rate (*RR* = 1.80, 95%*CI* = [1.37,2.38], *P *< .0001), and endoscopic cure rate (*RR* = 1.97, 95%*CI* = [1.36,2.86], *P *= .0004) of UC, but the adverse event rate (*RR* = 0.20, 95%*CI* = [0.01,4.00], *P* = .29) was similar. Trial sequential analysis indicated that the efficacy endpoint was conclusive. Harbord test confirmed no significant publication bias. The quality of evidence for these outcomes ranges from low to medium.

**Conclusion:**

The clinical efficacy of acupoint catgut embedding in the treatment of UC is superior to that of conventional western medicine, and the safety may be equivalent to that of conventional western medicine, which has the value of further research and exploration.

## 1. Introduction

Ulcerative colitis (UC) is a nonspecific chronic inflammatory disease of unknown etiology, which limited to the mucosa and submucosa of the colon and rectum.^[1]^ Epidemiology shows that the incidence of UC in Europe is 505/100,000, ranking first in the world. This is immediately followed by 248/100,000 in Canada and 214/100,000 in the United States. It is estimated that the prevalence in the Chinese population is about 11.6/100,000.^[2]^ Relevant reports pointed out that the incidence and prevalence of UC in developed countries in Europe and the United States have gradually stabilized, while the incidence and prevalence of non-developed countries in East Asia and South America have shown a significant upward trend, and UC has become a common and frequently-occurring disease worldwide.^[3,4]^ Abdominal pain, tenesmus, mucus, stool with mucus and blood are the typical symptoms of UC,^[5]^ and some may even accompany systemic symptoms or involve the skin, mucous membranes, eyes, joints, liver, gallbladder and other extraintestinal organs.^[6,7]^ It seriously endangers human health. Anti-inflammatory and immunomodulatory measures have delayed the progression of UC to a certain extent. However, adverse drug reactions such as nausea, vomiting, headache, anemia and recurrence after recovery are still difficult problems faced by clinicians.^[8,9]^ This urgently requires the intervention of novel therapeutic regimens.

Acupuncture is a traditional Chinese medicine therapy, and the guidelines suggest that acupuncture or the combination of acupuncture and medicine is an effective means to treat UC.^[10]^ Acupoint catgut embedding is a characteristic treatment method developed from acupuncture. Some studies have pointed out that acupoint catgut embedding has the characteristics of lasting, stable and precise curative effect,^[11]^ and can significantly improve intestinal inflammation,^[12]^ which may have a certain value in the treatment of UC. The efficacy of acupoint catgut embedding in the treatment of UC is controversial, and there has not been a systematic review comparing the efficacy of acupoint catgut embedding and that of conventional western medicine in the treatment of UC. Therefore, this study evaluated the clinical efficacy and safety of UC treated with acupoint catgut embedding by meta-analysis and trial sequential analysis, aiming to provide an evidence-based basis for clinical treatment.

## 2. Materials and methods

This meta-analysis and systematic review follows the Preferred Reporting Items for Systematic Reviews and Meta-Analyses guidelines.^[13]^ Because this study was based on a statistical analysis of the published literature, it was not necessary to review by an ethics committee or institutional board.

### 2.1. Search strategies

By searching VIP, Wanfang, China National Knowledge Infrastructure, China Biology Medicine, PubMed, Embase, Web of Science, the Cochrane Library and other databases, we searched for published clinical studies on acupoint catgut embedding in the treatment of UC. The publication time of the literature was limited from the time that the database was established to February 2022. Retrieval formula was: (acupoint catgut embedding OR catgut implantation at acupoint) AND (UC OR idiopathic proctocolitis OR colitis gravis). On this basis, we reviewed relevant reviews and latest clinical research reports on acupoint catgut embedding for UC, and supplemented relevant literature that may be missing.

### 2.2. Selection criteria

1. Study Design: randomized controlled trial.
2. Participants: meet the basic diagnosis of UC,^[2]^ and the baseline data are comparable.
3. Intervention and Comparison: The patients in the experimental group were treated with acupoint catgut embedding. The patients in the control group were treated with conventional western medicine (sulfasalazine, mesalazine, etc). And the 2 groups had the same course of treatment.
4. Outcome Indicators: The total effective rate of clinical symptoms, and the endoscopic total effective rate, the clinical symptom cure rate, the endoscopic cure rate were used as curative effect indexes, and the adverse event rate was used as safety indicators.

### 2.3. Exclusion criteria

1. Review, animal experiments, case reports, etc.
2. Repeated published research data.
3. Included subjects have other serious diseases.
4. Courses of treatment between the experimental group and the control group was inconsistent.

### 2.4. Literature selection

The relevant literature was obtained according to the retrieval method, and 2 researchers independently screened the literature according to the inclusion criteria, and any objections were adjudicated by a third party. First, duplicate literature was eliminated, and then the titles and abstracts were read to screen out case reports, animal experiments, reviews and other literature, and finally the full text was reviewed to determine the included literature.

### 2.5. Data extraction

A pre-designed statistical table was used to record the basic data of each study, including the first author and year, research center, sample size, intervention measures, course of treatment, and outcome indicators.

### 2.6. Risk of bias assessment and quality of selected studies

The risk of bias assessment tool recommended by the Cochrane Collaboration was used. The quality of the literature was assessed according to randomization method, allocation concealment, intervention blinding, outcome blinding, data integrity, selective reporting, and other biases. GRADEpro3.6 software was adopted in order to evaluate the quality of evidence for outcome indicators, and the evaluation factors included study limitations, inconsistency, indirectness, imprecision, and publication bias.

### 2.7. Statistical analysis

Revman5.3 was used to carry out meta-analysis. Continuous variables were used with mean difference and 95% confidence interval (95% CI) as effect statistics. Dichotomous variables were used with relative risk (RR) and 95% CI as effect statistics.

The level of heterogeneity was based on *I*^2^ test and Q test. If *I*^2^ < 50% and *P* > .1, the heterogeneity among studies was small, and the fixed effect model was used for analysis. Otherwise, random effects model analysis was used.

Trial sequential analysis (TSA) 0.9.5.10 Beta was used to carry out trial sequential analysis. If the cumulative Z value exceeds the TSA threshold or reaches the expected information value, the observed results of the current information volume were conclusive.

### 2.8. Publication bias

Stata15.0 was utilized to carry out Harbord regression to evaluate publication bias. If the hypothesis test shows that *P* > .1, there was no significant publication bias.

## 3. Results

### 3.1. Literature search results

**Figure 1.**
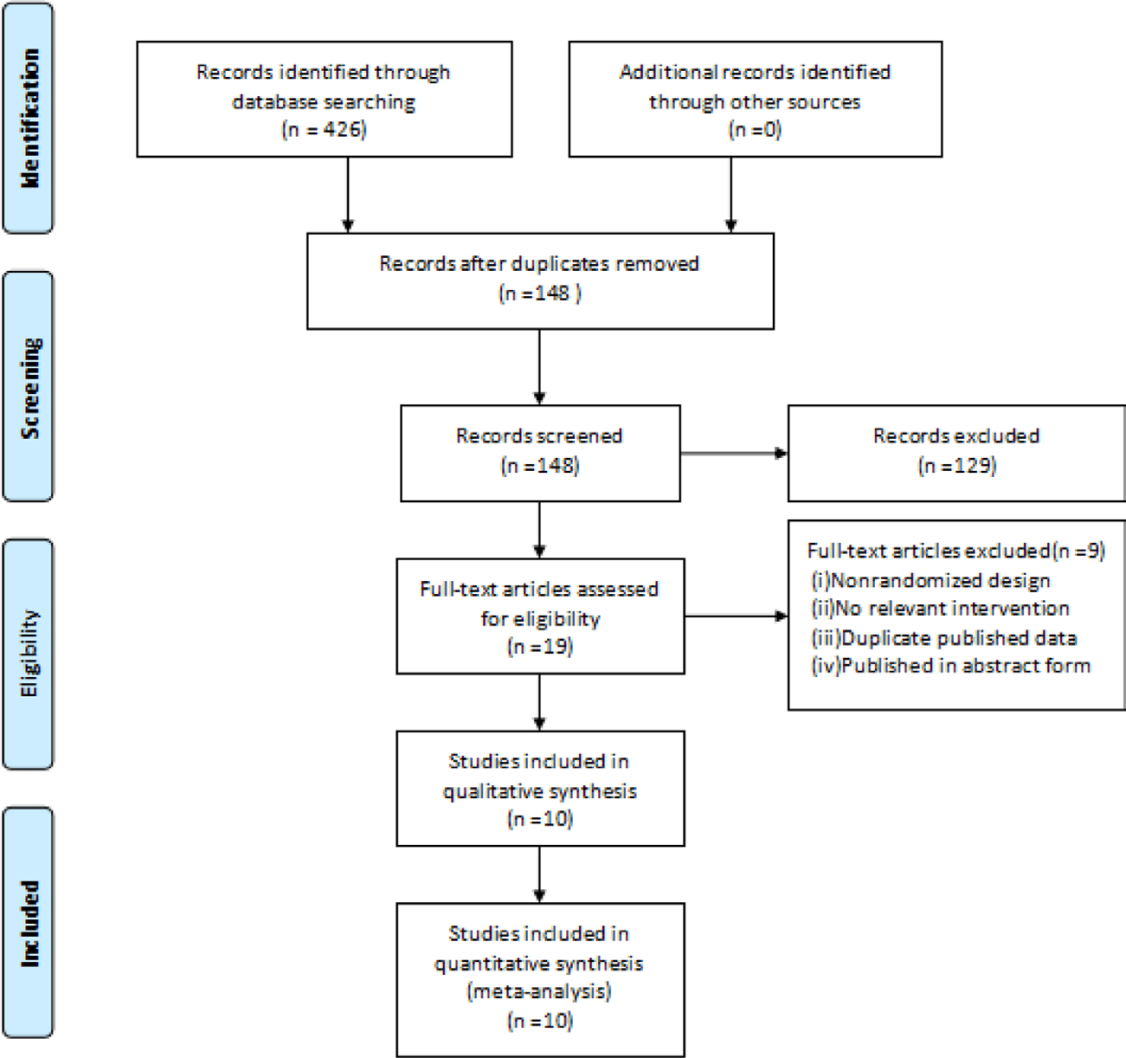
The flow chart of literature search and screening.

A total of 426 studies were detected, and 10 studies were finally included after culling and layer-by-layer screening.^[14–23]^ All studies were conducted in Chinese, with a total sample size of 782 cases, 450 cases in the experimental group and 332 cases in the control group. The screening process is shown in Figure 1 (For the original data statistics, please refer to Supplementary Material 1, http://links.lww.com/MD/H462).

### 3.2. Basic characteristics of included studies

A total of 10 studies were included,^[14–23]^ with the years from 2003 to 2020. All studies were published in full text. The research centers were all in China, and the course of treatment ranged from 1 to 6 months. The basic characteristics are shown in Table 1.

**Table 1:** Basic characteristics of the included studies.

Author and yr	Group	Usage and dosage	Sample size	Male/female	Age/yr	Course of disease/yr	Illness	Course of treatment/wk	Outcomes
ZHU Y 2003^[14]^	Acupoint catgut embedding	q14d	50	31/19	36.6	9.5	Low: 21	4	③⑤
							Moderate: 29		
	Sulfasalazine	po 1g qid	20	14/6	35.8	8.2	Low: 9		
							Moderate: 11		
CHEN J 2004^[15]^	Acupoint catgut embedding	q28d	100	62/38	42.5	/	/	24	①②③④
	Sulfasalazine	po 0.5g qid	30	18/12	40.2	/	/		
LI HJ 2006^[16]^	Acupoint catgut embedding	q28d	56	25/31	37.1	/	/	4	①②③④
	Sulfasalazine	po 4-6g/d, 2-3 times/d	60	27/33	37.3	/	/		
ZHU Y 2007^[17]^	Acupoint catgut embedding	q14d	30	14/16	36.6	9.5	Low: 11	4	③⑤
							Moderate: 16		
							Severe:3		
	Sulfasalazine	po 1g qid	30	15/15	35.8	8.2	Low: 10		
							Moderate: 6		
							Severe:4		
JI XL 2010^[18]^	Acupoint catgut embedding	q28d	35	18/17	41.23	4.8	/	24	③④
	Sulfasalazine	po 2g bid	20	11/9	55.48	5.1	/		
XU Y 2013^[19]^	Acupoint catgut embedding	/	35	20/15	34.3	3.91	Low: 19	8	①②③④
							Moderate: 16		
	Sulfasalazine	po 1g bid	30	17/13	35.5	4.2	Low: 17		
							Moderate: 13		
ZONG W2015^[20]^	Acupoint catgut embedding	/	20	/	/	/	/	6	①②③④
	Mesalamine	po 1g qid	20	/	/	/	/		
YANG Q 2019^[21]^	Acupoint catgut embedding	/	30	18/12	40.19	10.88	Low: 8	12	①②③④
							Moderate: 15		
							Severe:7		
	Sulfasalazine	po 1g qid	30	14/16	38.67	11,25	Low: 5		
							Moderate: 19		
							Severe:6		
GONG H 2020^[22]^	Acupoint catgut embedding	/	50	27/23	36.2	2.3	/	6	①②③④
	Mesalamine	po 1g qid	50	30/20	39.5	2.6	/		
LI W 2020^[23]^	Acupoint catgut embedding	q7d	30	17/13	45.38	1.84	/	6	③⑤
	Mesalamine	po 1g qid	30	16/14	46.22	2.18	/		

① represents the clinical symptom cure rate; ② represents the endoscopic cure rate; ③ represents the total effective rate of clinical symptoms; ④ represents the endoscopic total effective rate; ⑤ represents the adverse event rate.

### 3.3. Risk of bias assessment

**Figure 2.**
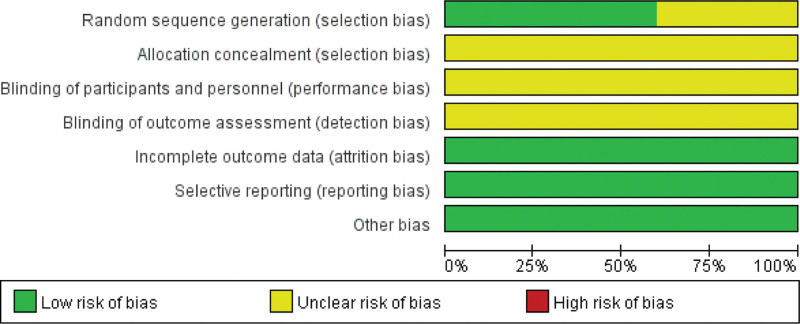
Risk of bias graph.

**Figure 3.**
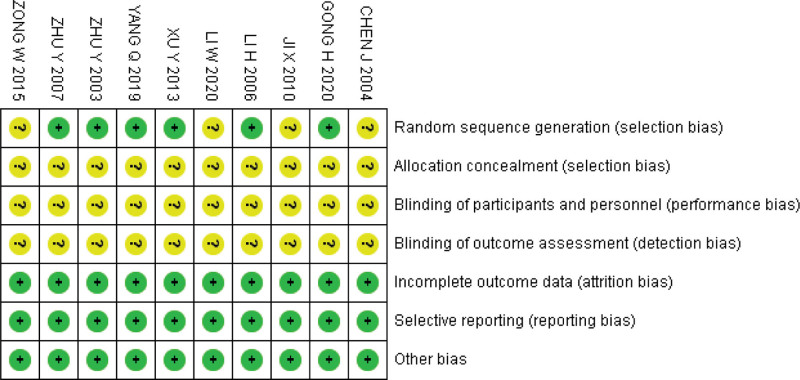
Risk of bias summary.

The Cochrane Bias Assessment Tool was used to assess the quality of the studies. The results showed that: 6 studies clearly defined randomization schemes, none of the studies described hidden schemes, none of the studies described intervention blinding and measurement blinding, none of the studies had significant dropout, and none of the studies had selective reporting, as shown in the Figures 2 and 3.

### 3.4. Evaluation of the quality of evidence

GRADEprofiler3.6 was used to assess the quality of evidence for the outcome indicators. The results showed that the quality of evidence for the total effective rate of clinical symptoms and the endoscopic total effective rate was medium, the quality of evidence for the clinical symptom cure rate, endoscopic cure rate, and the adverse event rate was low, and the strength of the recommendation was weak (Table 2).

**Table 2:** Evidence quality evaluation table for acupoint catgut embedding in the treatment of UC.

Outcomes	Limitation	Inconsistency	Indirectness	Imprecision	Publication bias	Effect size RR (95%CI)	Evidence level
Total effective rate of clinical symptoms	Serious	No serious	No serious	No serious	None	1.16 (1.09,1.24)	Moderate
Endoscopic total effective rate	Serious	No serious	No serious	No serious	None	1.16 (1.08,1.25)	Moderate
Clinical symptom cure rate	Serious	No serious	No serious	Serious	None	1.80 (1.37,2.38)	Low
Endoscopic cure rate	Serious	No serious	No serious	Serious	None	1.97 (1.36, 2.86)	Low
Adverse event rate	Serious	No serious	No serious	Serious	None	0.20 (0.01,4.00)	Low

95% CI = 95% confidence interval, RR = relative risk, UC=ulcerative colitis.

### 3.5. Meta-analysis

#### 3.5.1. Total effective rate of clinical symptoms.

**Figure 4.**
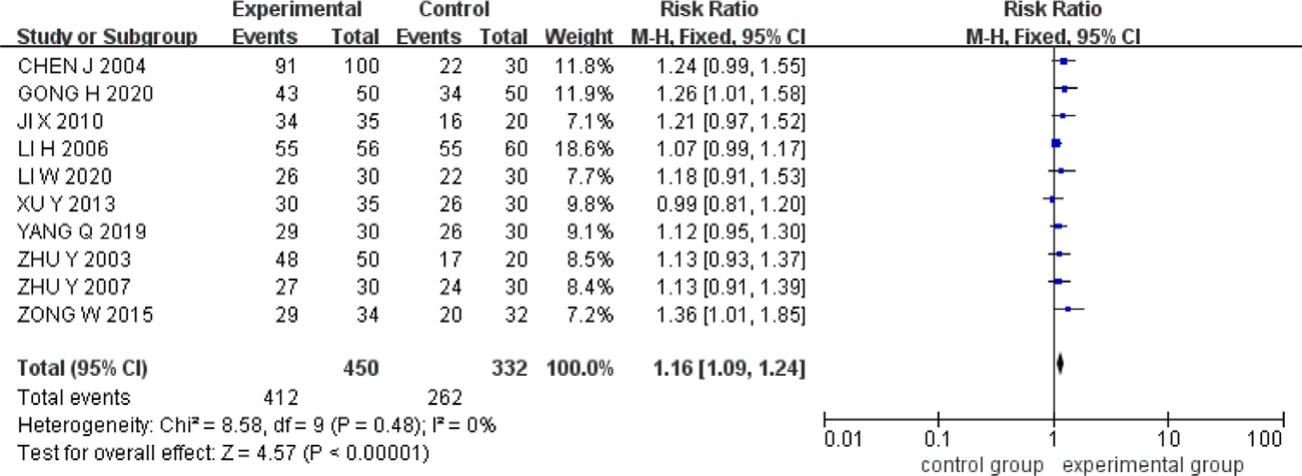
Forest plot of meta-analysis of total effective rate of clinical symptoms of acupoint catgut embedding in the treatment of UC. UC = ulcerative colitis.

Ten studies were included.^[14–23]^ The Q test and *I*^2^ test indicated that the heterogeneity was small (*P* = .48, *I*^2^ = 0%). The fixed effect model was used for analysis. The results showed that the total effective rate of clinical symptoms of acupoint catgut embedding was higher than that of conventional western medicine in the treatment of UC (*RR* = 1.16, *95%CI* = [1.09,1.24], *P < *.00001) (Fig. 4).

#### 3.5.2. Endoscopic total effective rate.

**Figure 5.**
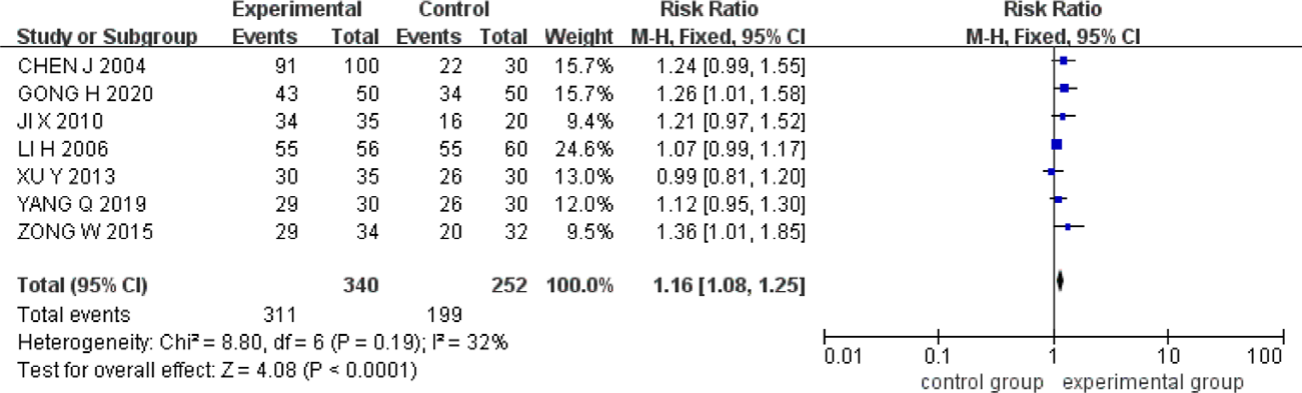
Forest plot of meta-analysis of endoscopic total effective rate in acupoint catgut embedding in the treatment of UC. UC = ulcerative colitis.

Seven studies were included.^[15,16,18–22]^ The Q test and *I*^2^ test indicated that the heterogeneity was small (*P* = .19, *I*^2^ = 32%). A fixed effect model was used for analysis. The results showed that the endoscopic total effective rate of acupoint catgut embedding was higher than that of conventional western medicine in the treatment of UC (*RR* = 1.16, *95%CI* = [1.08,1.25], *P < *.0001) (Fig. 5).

#### 3.5.3. Clinical symptom cure rate.

**Figure 6.**
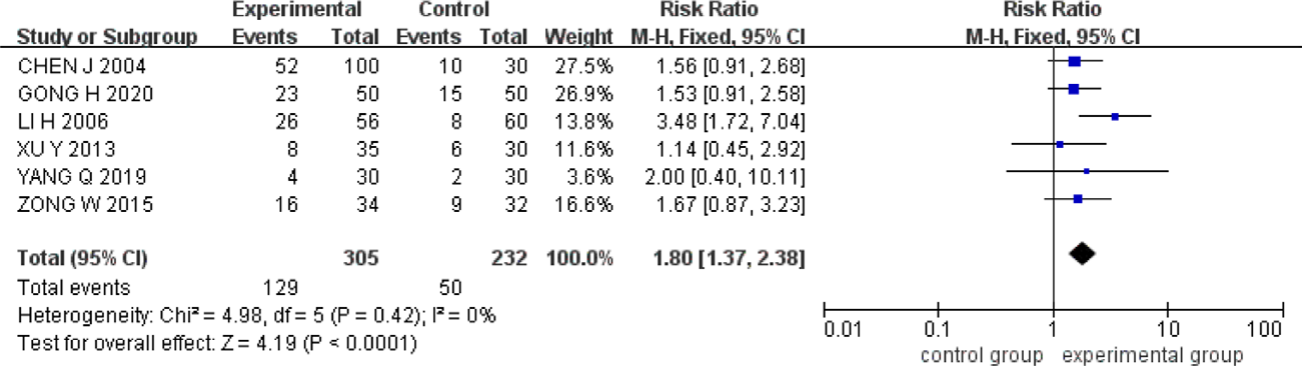
Forest plot of meta-analysis of clinical symptom cure rate of acupoint catgut embedding in the treatment of UC. UC = ulcerative colitis.

Six studies were included.^[15,16,19–22]^ The Q test and *I*^2^ test indicated that the heterogeneity was small (*P* = .42, *I*^*2*^ = 0%). A fixed effect model was used for analysis, and the results showed that the clinical symptom cure rate of acupoint catgut embedding was higher than that of conventional western medicine in the treatment of UC (*RR* = 1.80, *95%CI* = [1.37,2.38], *P < *.0001) (Fig. 6).

#### 3.5.4. Endoscopic cure rate.

**Figure 7.**
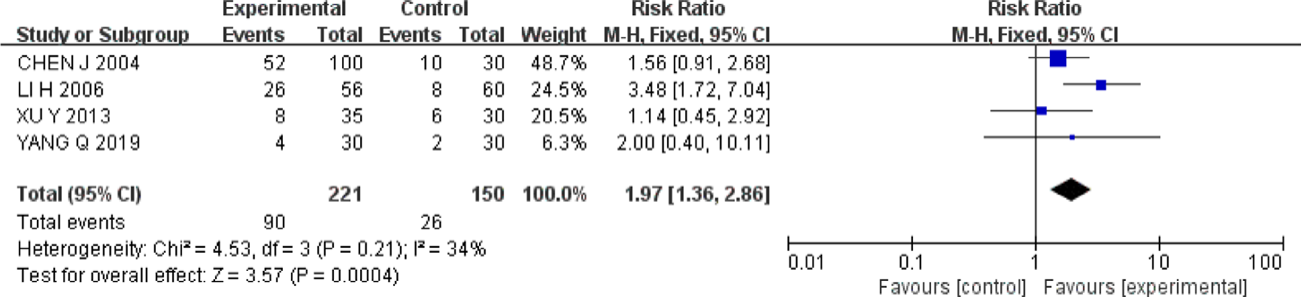
Forest plot of meta-analysis of endoscopic cure rate of acupoint catgut embedding in the treatment of UC. UC = ulcerative colitis.

Four studies were included.^[15,16,19,21]^ The Q test and *I*^2^ test indicated that the heterogeneity was small (*P* = .21, *I*^*2*^ = 34%). A fixed effect model was used for analysis, and the results showed that the endoscopic cure rate of acupoint catgut embedding was higher than that of conventional western medicine in the treatment of UC (*RR* = 1.97, *95%CI* = [1.36,2.86], *P* = .0004) (Fig. 7).

#### 3.5.5. Adverse event rate.

**Figure 8.**
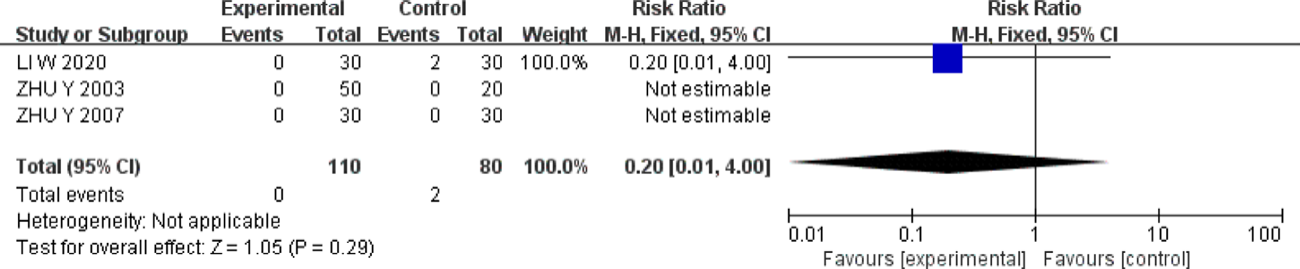
Forest plot of meta-analysis of the adverse event rate of acupoint catgut embedding in the treatment of UC. UC = ulcerative colitis.

Four studies were included.^[14,17,20,23]^ The Q test and *I*^2^ test indicated that the heterogeneity was small (*P* = 1.00, *I*^*2*^ = 0%). A fixed effect model was used for analysis. The results showed that the adverse event rate of acupoint catgut embedding of acupoint catgut embedding was comparable with that of conventional western medicine in the treatment of UC (*RR* = 0.20, *95%CI* = [0.01,4.00], *P* = .29) (Fig. 8).

### 3.6. Subgroup analysis

**Figure 9.**
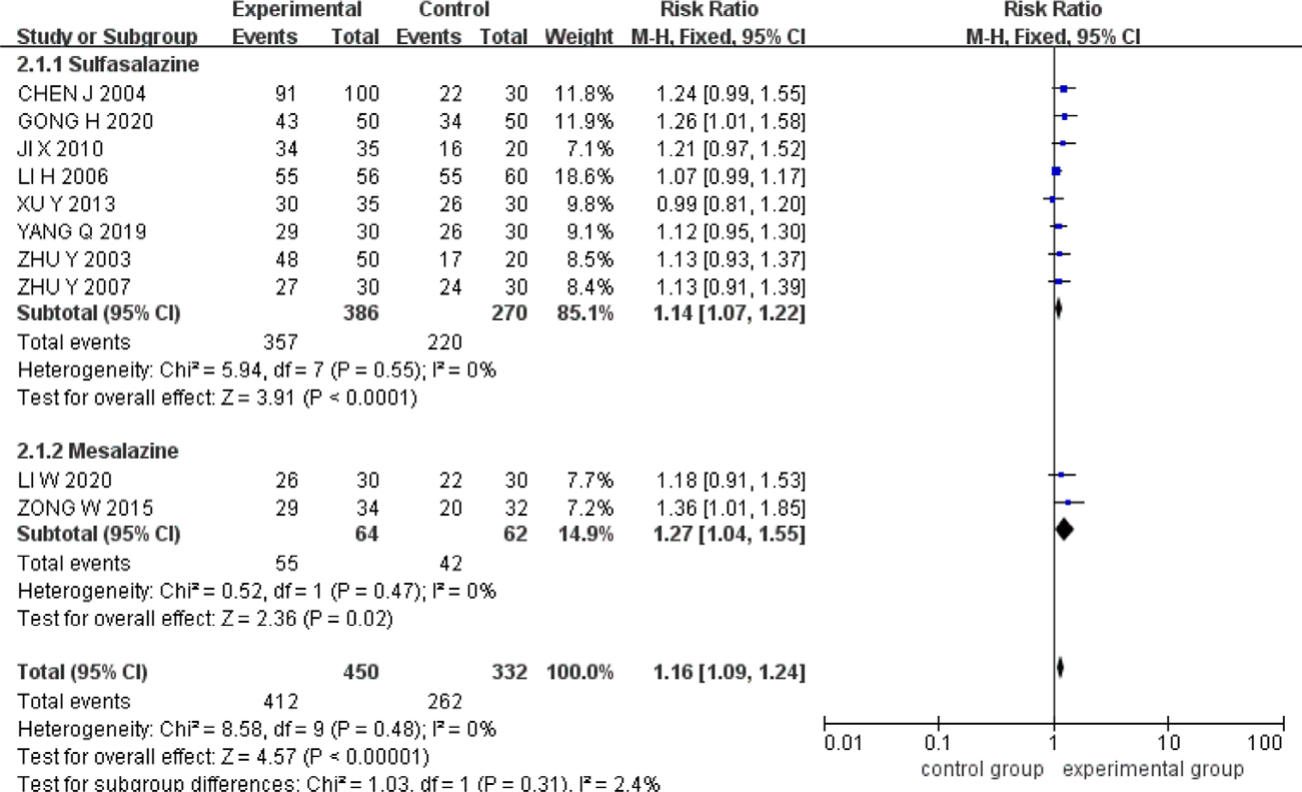
Drug subgroup analysis of acupoint catgut embedding in the treatment of UC. UC = ulcerative colitis.

Taking the total effective rate of clinical symptoms as the index, subgroups were established with sulfasalazine and mesalazine as the subjects, and the differences in the therapeutic effects between acupoint catgut embedding and the 2 drugs were compared. The results showed that the clinical efficacy of acupoint catgut embedding was better than that of sulfasalazine (*RR* = 1.14, *95%CI* = [1.07,1.22], *P < *.0001) and mesalazine (*RR* = 1.27, *95%CI* = [1.04,1.55], *P* = .02) (Fig. 9).

**Figure 10.**
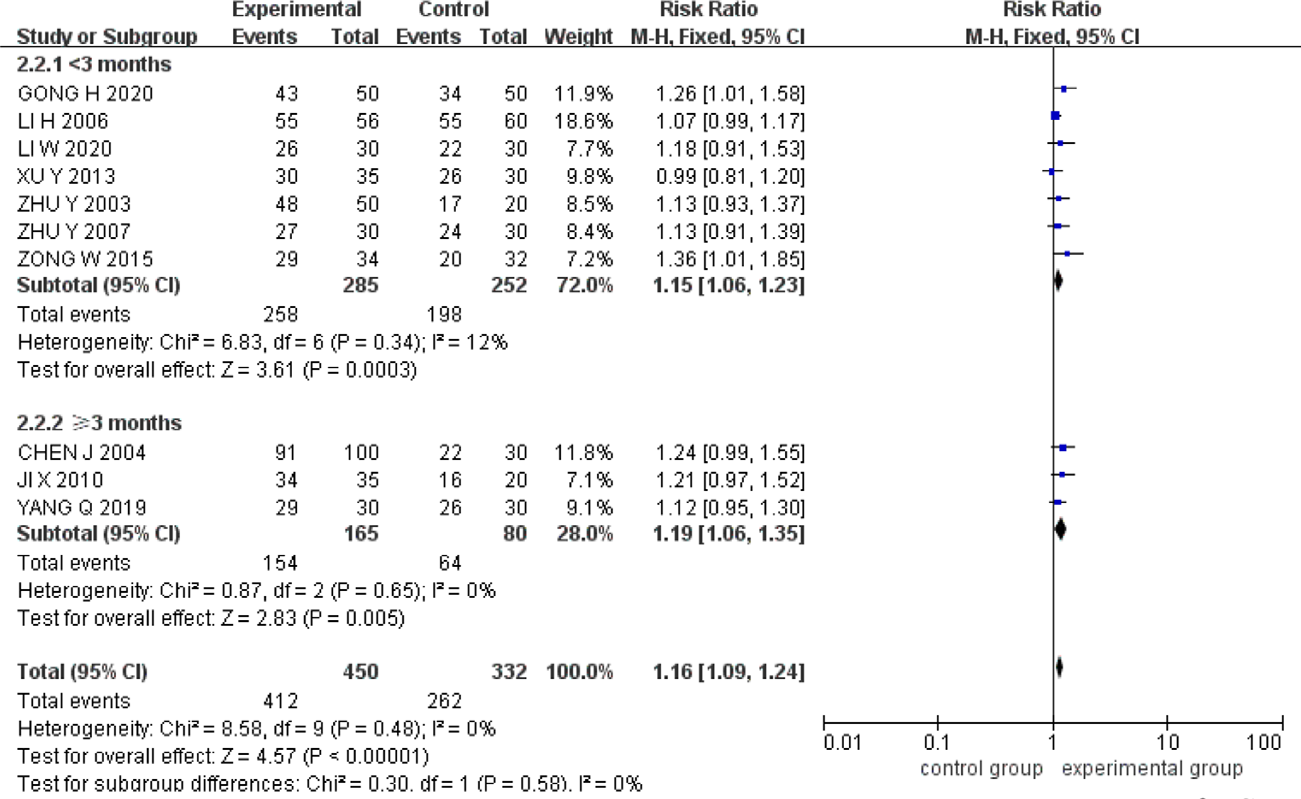
Subgroup analysis of the course of acupoint catgut embedding in the treatment of UC. UC = ulcerative colitis.

Taking the total effective rate of clinical symptoms as the index, subgroups were established with short courses of treatment (≤3 months) and long courses of treatment (>3 months), and the differences in the efficacy of acupoint catgut embedding and conventional western medicine under different courses of treatment were compared. The results are outstanding, the efficacy of acupoint catgut embedding in short courses (*RR* = 1.15, 95%*CI* = [1.06,1.23], *P* = .0003) and long courses (*RR* = 1.19, 95%*CI* = [1.06,1.35], *P* = .005) is better than that of conventional western medicine (Fig. 10).

### 3.7. Trial sequential analysis

**Figure 11.**
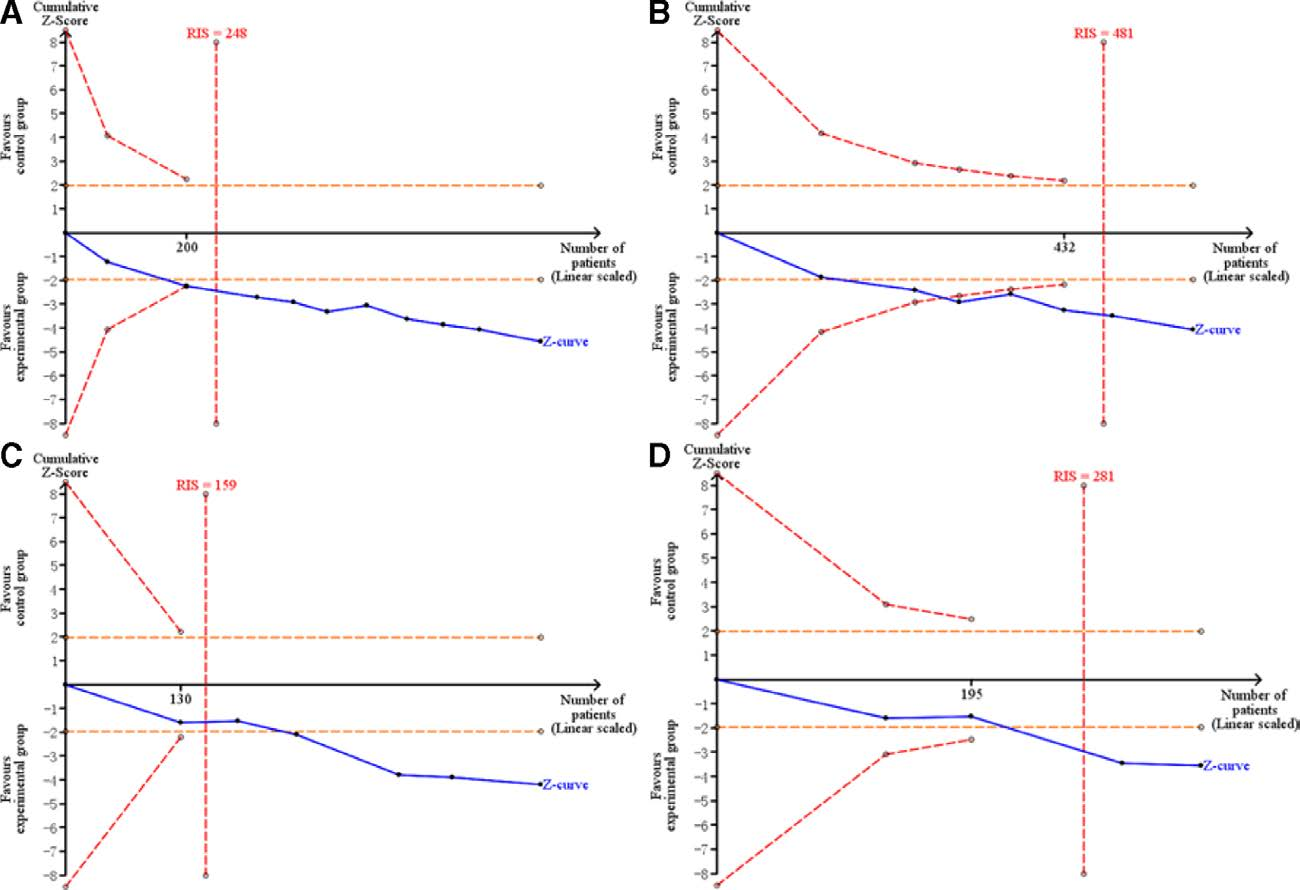
Trial sequential analysis of acupoint catgut embedding in the treatment of UC. UC = ulcerative colitis.

The probability of Type I error was set as α = 0.05, and the probability of Type II error was set as β = 0.20. The positive rate of the experimental group and the control group were calculated using the included data. RIS was set to the expected information value. The results showed that the cumulative Z value of total effective rate of clinical symptoms, endoscopic total effective rate, clinical symptom cure rate and endoscopic cure rate crossed the RIS threshold (Fig. 11). It indicated that the observed results of the current amount of information are conclusive. Since the number of events in the trial group in the adverse event rate data is 0, the trial sequential analysis cannot be carried out, and the safety endpoint needs to be demonstrated by more studies.

### 3.8. Publication bias

**Figure 12.**
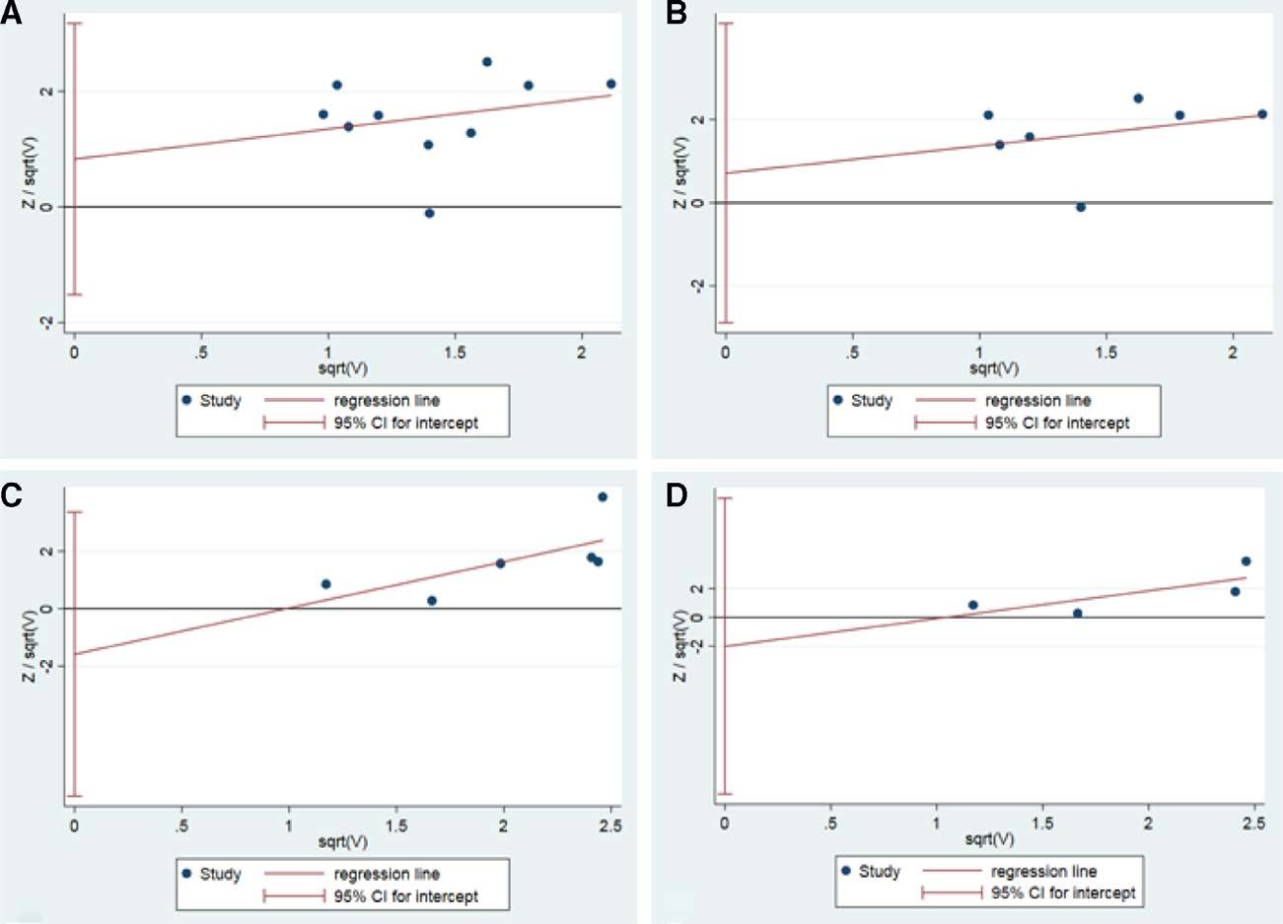
Harbord regression of acupoint catgut embedding of UC. UC = ulcerative colitis.

The Harbord linear regression method was used to evaluate publication bias, and the results showed that there was no significant publication bias in the total effective rate of clinical symptoms (*P* = .44), and endoscopic total effective rate (*P* = .63), clinical symptom cure rate (*P* = .42), endoscopic cure rate (*P* = .49) (Fig. 12).

## 4. Discussion

In recent years, more and more scholars have devoted themselves to the research of UC, but there is still no specific drug for the treatment of this disease. Although conventional drugs such as sulfasalazine and mesalazine can control inflammation in the colorectum to some extent, the financial burden, drug tolerance, and decreased compliance that accompany long-term drug use continue to plague clinicians and patients.^[24]^ Acupoint catgut embedding is a characteristic therapy developed based on the theory of acupuncture and moxibustion in traditional Chinese medicine. It is designed to stimulate the body to adjust and repair through the gentle and persistent stimulation of acupuncture points, with the intention of stimulating positive energy and regulating yin and yang. It has been applied with definite efficacy in a variety of diseases such as insomnia, obesity, osteoporosis and irritable bowel syndrome,^[11, 25–27]^ and also has a considerable effect in the treatment of UC.^[12]^ In addition, the majority of scholars recommend a period of 1 to 2 weeks for acupoint catgut embedding to replace lines. Furthermore, acupoint catgut embedding is a simple and easy operation, which means that the program may have better compliance, and at the same time help to reduce the development of drug tolerance and reduce the financial burden on patients.

### 4.1. Analysis of results

Meta-analysis results showed that acupoint catgut embedding increased the clinical symptom cure rate by 80% and endoscopic cure rate by about 97%, and the total effective rate of clinical symptoms and endoscopic total effective rate by 16% compared with conventional western medicine in the treatment of UC. The above results are still established after TSA correction, which is conclusive. This suggests that the clinical efficacy of acupoint catgut embedding in the treatment of UC is better than that of conventional western medicine. Subgroup analysis showed that acupoint catgut embedding improved the total effective rate of clinical symptoms by 14% relative to sulfasalazine and 27% relative to mesalazine, suggesting that the clinical efficacy of acupoint catgut embedding was superior to that of sulfasalazine and mesalazine. Subgroup analysis also showed that the treatment of short courses (≤3 months) and long courses (>3 months) with acupuncture points provided additional benefits compared to conventional western medicine. In terms of safety, the results of the meta-analysis suggested that the adverse event rate to acupuncture versus conventional western medicine for UC was comparable. In fact, in the data published by the Food and Drug Administration, the rate of headache induced by mesalazine was 14% and the rate of nausea induced by sulfasalazine was 19%, whereas no significant adverse effects have been reported in the treatment of UC with acupoint catgut embedding. Based on the above evidence, we can speculate that the risk of adverse event with acupoint catgut embedding may be lower than with conventional western medicine, and that the negative results of the meta-analysis may be due to insufficient sample size.

### 4.2. Analysis of mechanisms

The mechanism of acupoint catgut embedding for UC may be related to the inhibition of inflammatory response, induction of apoptosis and enhancement of specific immunity. Firstly, acupoint catgut embedding can inhibit the inflammatory response. Some studies have shown that acupoint catgut embedding thread exerts rapid and long-lasting anti-inflammatory effects and mucosal repair by upregulating β2 adrenergic receptor expression and blocking interleukin (IL)-17 and nuclear factor kB p65 expression in rat spleen lymphocytes,^[28]^ and is also able to reduce 5-lipoxygenase expression,^[20]^ increase the level of the flat anti-inflammatory factor IL-2,^[29]^ and reduce the levels of the pro-inflammatory factors IL-6, IL-8 and tumor necrosis factor-α.^[20,22]^ Secondly, acupoint catgut embedding can induce apoptosis. It has been reported that acupoint catgut embedding method when applied to UC can increase the expression of apoptosis-inducing genes Apo-1 and Apo-2.7, decrease the expression of apoptosis-inhibiting gene Bcl-2 and promote lymphocyte apoptosis, which in turn inhibits inflammation in the intestinal mucosa and submucosa.^[17]^ This report also indicated that acupoint catgut embedding increased the expression of apoptosis-inducing genes and decreased the expression of apoptosis-inhibiting genes better than that of sulfasalazine, and that there was a synergistic effect between acupoint catgut embedding and sulfasalazine.^[17]^ Thirdly, acupoint catgut embedding can boost the body’s immunity. It was found that acupoint catgut embedding significantly increased the cluster of differentiation (CD) 4 percentage, CD8 percentage and CD4/CD8 in patients with UC, achieving an increase in the body’s immunity by increasing CD4 activity.^[14]^ It has also been suggested that CD44 and CD54 levels in the tissues of patients with UC treated with acupoint catgut embedding were significantly higher than before the treatment and were superior to those in the salbutamol group.^[29]^ This implies a positive effect of acupoint catgut embedding on the immune regulation of the body.^[29]^

### 4.3. Acupuncture point analysis

**Figure 13.**
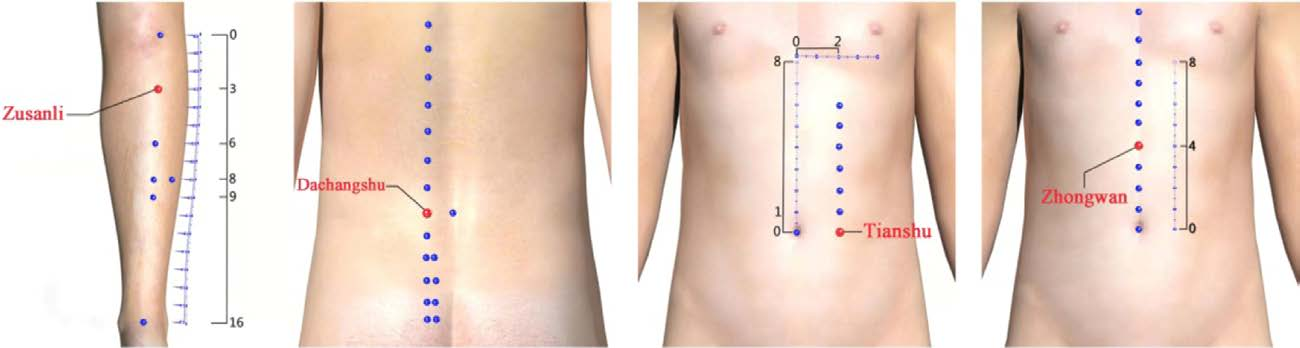
Schematic diagram of the core acupoints of acupoint catgut embedding in the treatment of UC. UC = ulcerative colitis.

Although meta-analyses have argued for the benefits of acupoint catgut embedding in the treatment of UC, the selection of acupoints for embedding remains somewhat controversial. A recently published data mining study sheds light on the issue of acupoint selection. The study analyzed 62 formulas from 35 studies over the last 15 years. And the results showed that the frequency analysis of acupoints found that Zusanli, Tianshu, Dachangshu and Zhongwan were the most commonly used acupoints, which application frequency exceeded 10%. Furthermore, Pishu 7.3%, Guanyuan 7.3%, Shangjuxu 6.6%, Qihai 3.8%, Shenshu 2.8%, and Sanyinjiao 2.1% were also more commonly used acupoints. The frequency analysis of the meridians suggested that 42.16% of the Wei meridian, 31.74% of the Pangguang meridian and 30.87% of the Ren meridian were the main meridians, while the remaining meridians were selected with a frequency of less than 5%. This data mining revealed that the most commonly used point combinations were Zusanli-Zhongwan-Tianshu and Zusanli-Dachangshu-Tianshu by point correlation analysis.^[30]^ In our study, the frequency of acupoints was analyzed in the 10 included papers, and the results showed that 90% (9/10) of the Zusanli, 90% (9/10) of the Dachangshu, 80% (8/10) of the Tianshu, 60% (6/10) of the Pishu, 60% (6/10) of the Guanyuan and 40% (4/10) of the Zhongwan were the main acupoints used for the treatment of UC with acupoint embedding, similar to the results of the data mining described above. In summary, we recommend that Zusanli, Dachangshu, Tianshu and Zhongwan are the core acupuncture points for the treatment of UC, and the schematic diagram of the acupuncture points is shown in Figure 13.

### 4.4. Limitations

The evaluation of the quality of evidence showed moderate quality of evidence for 2 indicators and low quality of evidence for 3 indicators, suggesting some limitations (below).

1. The original data for each study were from China and the study participants were all of Chinese descent, so the results may only apply to Chinese people, limiting the generalizability of the results.
2. There were differences in the acupuncture point protocols of the experimental groups, and the interventions are not highly consistent, which may have led to clinical heterogeneity.
3. All studies did not take a description of the concealment method, which may lead to selectivity bias.
4. All studies did not describe intervention blinding and measurement blinding, which may lead to implementation bias and outcome bias.
5. The data from studies on inflammatory indicators and apoptotic proteins at this stage of the study were small and not yet sufficient for meta-analysis.

### 4.5. Suggestions for future research

Based on the results of the limitations analysis of this study, the following points should be noted in future RCT studies of acupoint catgut embedding for UC.

1. Scientific research methods: Randomized, concealed, intervention-blinded and measurement-blinded methods are fully utilized in the development of clinical studies. It is also important to clearly state in the thesis report in which form exactly which research methods were used to improve the quality of the literature and the reliability of the study.
2. Consistency of interventions: Both the type and number of acupuncture points can affect the reliability of the analysis results, so it is important to achieve consistency in the matching of acupuncture points wherever possible to improve the credibility of the study.
3. Focus on studies of inflammatory indicators and apoptotic proteins: The existing clinical studies of acupoint catgut embedding for UC have less data on inflammatory indicators and apoptotic proteins, which should be supplemented in future trials to provide more clinical evidence for evidence-based analysis.

## 5. Conclusion

The clinical efficacy of acupoint catgut embedding for UC is superior to that of conventional western medicine, and the safety may be equivalent to that of conventional western medicine, which has the value of further research and exploration.

## Author contributions

The idea of this research was proposed and conceived by Yunfeng Yu. This study was also completed with of Manli Zhou, Yaling Tong, Shuang Yin, Gang Hu, and reviewed and edited by Weixiong Jian, Ying Zhu.

**Data curation:** Shuang Yin.

**Methodology:** Manli Zhou.

**Software:** Yaling Tong.

**Supervision:** Gang Hu.

**Writing – original draft:** Yunfeng Yu.

**Writing – review & editing:** Weixiong Jian, Ying Zhu.

## Supplementary Material
